# Meaningful Activities and Recovery (MA&R): a co-led peer occupational therapy intervention for people with psychiatric disabilities. Results from a randomized controlled trial

**DOI:** 10.1186/s12888-023-04875-w

**Published:** 2023-06-06

**Authors:** Siv-Therese Bogevik Bjørkedal, Ulrika Bejerholm, Carsten Hjorthøj, Tom Møller, Lene Falgaard Eplov

**Affiliations:** 1 CORE: Copenhagen Research for Mental Health, Gentofte Hospitalsvej 15, 3A, 2900 Hellerup, Denmark; 2 Department of Health Sciences, Centre of Evidence‐Based Psychosocial Interventions, CEPI, Lund University, Lund, Sweden; 3 Department of Research, Development and Education, Division of Psychiatry and Habilitation, Region Skåne, Lund, Sweden; 4 University Hospitals Centre for Health Research (UCSF), Department 9701, Copenhagen University Hospital, Rigshospitalet, Blegdamsvej 9, 2100 Copenhagen, Denmark; 5 Department of Public Health, University of Copenhagen, Øster Farimagsgade 5, 1353 Copenhagen K, Denmark

**Keywords:** Rehabilitation, Peer support, Community mental health, Evaluation, Psychiatric disabilities, Psychosocial intervention

## Abstract

**Background:**

Activity and participation are critical to health and wellbeing. Limited evidence exists on how to support people with mental illness in participating in everyday activities.

**Aim:**

To investigate the effectiveness of Meaningful Activities and Recovery (MA&R), a co-led peer occupational therapy intervention focusing on activity engagement, functioning, quality of life, and personal recovery.

**Methods:**

In a statistician blinded, multicenter RCT including 139 participants from seven community and municipal mental health services in Denmark, participants were randomly assigned to 1) MA&R and standard mental health care or 2) standard mental health care. The MA&R intervention lasted 8 months and consisted of 11 group sessions, 11 individual sessions, and support to engage in activities. The primary outcome, activity engagement, was measured using Profile of Occupational Engagement in People with Severe Mental Illness (POES-S). Outcomes were measured at baseline and post-intervention follow-up.

**Results:**

Meaningful Activities and Recovery was delivered with high fidelity and 83% completed the intervention. It did not demonstrate superiority to standard mental health care, as intention-to treat analysis revealed no significant differences between the groups in activity engagement or any of the secondary outcomes.

**Conclusion:**

We did not find positive effects of MA&R, possibly because of COVID-19 and related restrictions. Fidelity assessments and adherence rates suggest that MA&R is feasible and acceptable. However, future studies should focus on refining the intervention before investigating its effectiveness.

**Trial registration:**

The trial was registered 24/05/2019 at ClinicalTrials.gov NCT03963245.

**Supplementary Information:**

The online version contains supplementary material available at 10.1186/s12888-023-04875-w.

## Introduction

Psychiatric disabilities occur when mental health conditions and environmental barriers inhibit individuals in engaging in everyday activities such as work and civic life [1, 2]. These internal and external barriers may have severe social and personal consequences, including isolation, loneliness, loss of daily structure and social identities [3–6]. Occupational therapy is a professional health intervention based on the view that activity is fundamental to human health and wellbeing [7–10]. Comprising actual activity (activity performance) and reflection on the experience of activity, activity engagement has been linked to empowerment, sense of control, quality of life, and recovery [11–19]. Occupational therapists seek to enable activity engagement by enhancing people`s abilities and opportunities or by modifying their environments [20]. Interventions that target activity engagement should be developed and evaluated, as the evidence base informing occupational therapy practice in mental health is sparse [21–23]. In an randomized controlled trial (RCT), the group-based occupational therapy intervention Balancing Everyday Life (BEL) showed a small but significant effect on activity engagement [24]. Yet, results from a qualitative synthesis suggest that the group format may not be ideal for enabling activity engagement in the community [25]. A growing body of evidence [18, 26, 27] suggests that the experience of meaning in performing everyday activities is an important aspect of recovery processes. Hence, new approaches to supporting people in finding meaning and new ways to enable activity engagement are warranted. Combining peer workers’ lived experiences of mental illness and recovery with occupational therapists’ knowledge about the therapeutic use of activities holds potential for strengthening mental health practices that support recovery in the context of everyday life and for connecting service users to the community [28, 29].

We developed Meaningful Activities and Recovery (MA&R), a co-led peer occupational therapy intervention, to enable engagement in meaningful activities among people with psychiatric disabilities. The intervention was investigated in a multicenter, statistician-blinded RCT. The RCT compared the effectiveness of two interventions: 1) MA&R in addition to standard mental health care and 2) standard mental health care. We hypothesized that MA&R in addition to standard mental health care was more effective in improving activity engagement than standard mental health care alone when using the self-report version of the Profiles of Occupational Engagement in people with Severe Mental Illness (POES-S) instrument [30]. Hence, activity engagement was the primary outcome in this study. We also hypothesized that MA&R would be more effective in improving functioning, personal recovery and quality of life.

## Methods

The RCT methodology is described in detail in a protocol paper by Bjørkedal et al. [31]. No amendments were made after the paper was published.

### Participants

Eligible participants 1) were 18 years or older; 2) could speak and understand Danish; 3) provided informed consent and 4) had a psychiatric disability assessed by the primary researcher using MINI ICF Rating for limitation of Activities and Participation in Psychological Disorders (MINI-ICF-APP). In this study, psychiatric disability was considered if the participant scored 1 (mild impairment) or more in at least 1 of the 13 capacity domains (e.g. planning an structuring tasks) in the MINI ICF App [32].

### Setting

The study was conducted in three Danish cities in three community mental health centers (CMHCs), three activity and social support centers (ASSCs), and one rehabilitation team. In Denmark, public mental health services are organized into two sectors: the CMCHs that offer treatment to patients through inpatient and outpatient services [33] and municipal mental health services that offer social and rehabilitation services [34]. Both sectors serve citizens with mental illness in their catchment area. The sectors complement each other; therefore, participants often received services from both sectors. Meaningful Activities and Recovery was co-led by occupational therapists and peer workers employed at the various sites (the rehabilitation team and two ASSCs) or by first author, who is an occupational therapist and a peer worker employed in the project (the CMCHs and one ASSC).

### Interventions

#### MA&R

The intervention consisted of 22 sessions—11 group sessions and 11 one-to-one sessions that took place alternately. In the group sessions, participants were introduced to topics related to activities, health, recovery, and strategies for activity engagement. The group sessions were facilitated by the peer worker and the occupational therapist, who provided a combination of theoretical knowledge and experienced-based knowledge. The topics were introduced, using didactic presentations, reflective questions from the MA&R workbook, and peer exchange. Some group sessions also utilized other methods, such as storytelling and photovoice activities. In the one-on-one sessions, each participant met with the occupational therapists and the peer worker. These sessions provided opportunities for the participants to reflect on and discuss topics from the group sessions, such as the connection between activity engagement and states of flow. Group sessions typically lasted 90 min, including a break of 10–15 min, while one-on-one sessions typically lasted between 30 to 60 min [31].

In addition to the scheduled sessions, participants were also offered individual support to engage in activities. Individual support was optional and based on the participants’ wishes and goals. The support was flexible and could be provided in multiple ways, such as companionship, practical help, supportive conversations or help to create new strategies for activity engagement. For instance, a participant who wanted to exercise regularly in the gym was offered companionship by the occupational therapist to enhance a sense of commitment and build a routine around exercising. Another participant who wished to write a book was offered guidance and advice from the peer worker, who was an author[35]. No limitation was set on the amount of individual support provided to participants. For practical reasons, we anticipated that participants would have four encounters with either the occupational therapist or the peer worker.

Meaningful Activities and Recovery was organized into two modules: MA&R I with two weekly sessions focusing on exploring and recognizing meaningful activities, and MA&R II with two monthly sessions allowing participants to engage in new meaningful activities at their own pace. The intervention was delivered in facilities in the participating sites, in participants’ homes or in the community. Providers received training in the methods used beforehand (a 3-h workshop and individual preparation) and consecutive supervision (1,5 h × 6). Supervision was given by the first author, who developed MA&R.

Participants allocated to the MA&R intervention were also offered standard mental health care, as MA&R was investigated as an add-on to usual care in this RCT.

#### Standard mental health care.

**Fig. 1:**
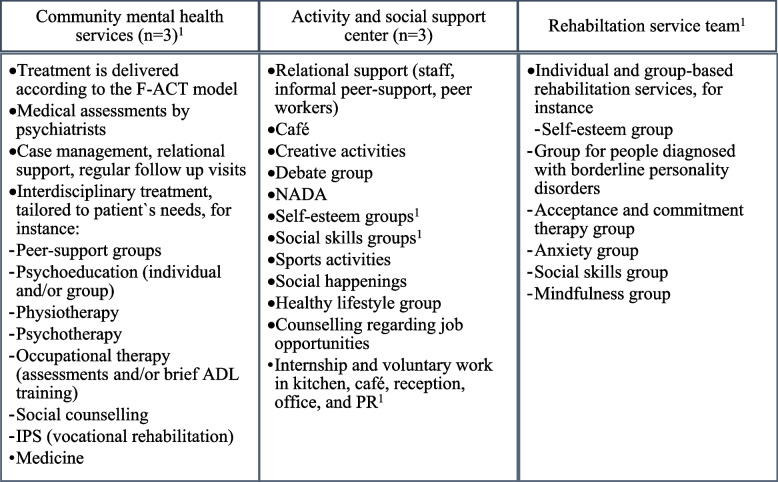
An overview over interventions included in standard mental health care, in the recruitment sites. ^1^Services that require referral and visitation

The treatment provided in CMCHs was the multidisciplinary Flexible Assertive Community Treatment model (the F-ACT model) [36]. In addition to F-ACT, standard mental health care included services shown in Fig. 1.

Standard mental health care offered at the ASSCs included relational support, cafés, group activities, and vocational rehabilitation. The rehabilitation team offered individual and group-based rehabilitation services (Fig. 1).

### Data collection and outcome measures

Sociodemographic and clinical self-report data were obtained at enrollment. The MINI-ICF-APP was used to measure psychiatric disability. This observer-rated instrument consists of a semi-structured interview guide covering 13 domains of capacity limitations, e.g., self-care and relationships, and gives a total score between 0 and 52. Higher value indicates more severe psychiatric disability [32]. The MINI-ICF-APP provides cut-off scores which define the degree of disability: 3–7 points indicates mild disability, 8–15 points moderate severity, 16 to 24 points marked disability, 25 to 37 points severe disability, and 38 to 52 points extreme disability [37].

Outcomes were measured twice, at baseline and at the end of the MA&R intervention.

The POES-S [30] was chosen to measure the primary outcome, activity engagement. Developed on the basis of time-use diary studies of persons with schizophrenia, POES-S is a self-report version of POES [11, 38] and has been found to be a valid and reliable instrument for measuring activity engagement [38–40]. Secondary and exploratory outcomes were functioning, personal recovery, and quality of life. An overview of outcomes and description of outcome measures are found in Table 1.

**Table 1:** Overview over outcomes and brief description of outcome measures

Outcome	Measure	Description	Number of items	Range
*Primary outcome*				
Occupational engagement	Profiles of Occupational Engagement in people with Severe mental illness-Self-rated version (POES-S). (140)	POES- S assess time-use patterns of activity performance and the extent to which these patterns are characterized by engagement. The measure consists of two parts: a 24-h, yesterday time-use diary sheet, and a questionnaire with nine items related to occupational engagement. The nine items concern: 1) daily rhythm of activity and rest, 2) moving around in society without hinderance, 3) variety and range of occupations, 4) spending time in a variety of social environments without hinderance, 5) social interplay, 6) making sense of occupational experiences 7) extent of meaningful occupations, 8) routines and 9) initiating performance. Each item is rated on a scale from 1 to 4. Items are added to a total score, higher values indicate greater occupational engagement.(140)	9	9–36
*Secondary outcomes*				
Functioning	WHODAS 2.0 12-item version. (141)	WHODAS 2.0 12 item version consists of 12 items derived from the WHODAS 2.0 36-item version, covering six domains of functioning: Cognition, mobility– moving & getting around, self-care, getting along– interacting with other people, life activities– domestic responsibilities, leisure, work & school, and participation– joining in community activities. Each item is rated on a scale from 1 to 5. The items can be added together to a sum score or converted into a complex score (using WHODAS software system) on a metric range.(141)	12	12–60 (or 0–100, metric range)
Personal recovery	Questionnaire about processes of recovery (QPR)(142)	QPR contains 15 items reflecting aspects of the personal recovery process, e.g., relationships, sense of agency and hope. Each item is rated on a scale from 0–4, and added together to a sum score. (142)	15	0–60
Quality of life	Manchester short assessment of quality of life (MANSA)(143)	MANSA contains 16 items related to quality of life, e.g., satisfaction with life, relationships, financial situation, etc. 12 of the items are numerical variables and be rated on a scale from 1–7 and added together to a total score. (143)		12–84
*Exploratory outcomes*				
Functioning	WHODAS 2.0 36 items version (141)	WHODAS 2.0. 36 item version consist of 36 items, covering the forementioned six domains of functioning. Each domain contains between five to eight items, each item is rated on a scale from 1 to 5 The items can be added together to a domain score and a sum score. The sum score can be converted into a complex score (using WHODAS software system) on a metric range. (141)	36	36–180 (or 0–100, metric range)
Health -related quality of life	EuroQol (EQ-5D-3L) (144)	EQ-5D-3L contains five dimensions: mobility, self-care, usual activities, depression/anxiety, and pain. Each combination of answers is converted and provided with a number between 0 and 1. It also entails a visual analogue scale (VAS) on self-rated health (1–100) (144)		0–1 (VAS scale 0–100)

### Harms

At post-intervention follow-up, we obtained number of admissions and bed days (psychiatric and somatic), number of deaths and causes, and measures on The Clinical Global Impression – Severity of Illness Scale (CGI-S).

### Sample size

Sample size calculation on primary outcome was performed. Using data from the BEL trial, [24] we assumed the standard deviation of the POES-S in the study population to be 6. The study had to include 128 participants, 64 in each group, to achieve a statistical power of 80% and a significance level of 5% and to detect a difference of 3 points corresponding to a moderate effect size, on the POES-S.

### Randomization

The participants were randomly assigned (1:1) to MA&R in addition to standard mental health care or standard health care alone. Randomization was performed by the primary researcher, who enrolled participants and collected the baseline data, using REDCap (Research Electronic Data Capture) [41, 42]. The allocation sequence was stratified by sex and used varying block sizes. To keep it unknown, the sequence was generated by a staff member external to the research team and stored away from the research team during the study period.

### Blinding

Researchers were blinded to the participants’ allocation during data collection, during analysis, and while writing the conclusion of the study. Participants who had help from a research assistant to complete questionnaires at follow-up were instructed not to reveal their intervention allocation to the research assistant. It was not possible to blind participants or the professionals to the intervention allocation.

### Fidelity assessment

An MA&R fidelity scale was developed to assess the delivery of the intervention. Fidelity was assessed by individual structured interviews with the providers and focus group interviews with participants All participants in MA&R were invited to the interviews. The MA&R fidelity scale covered six major components, all considered essential for successful intervention delivery: staffing, organization, group sessions, individual sessions, contact, and individual support. The scale ranged from 0 to 41. A fidelity score of 25 or more indicated that MA&R was delivered. Higher scores reflected higher degree of fidelity in the delivery of MA&R.

### Statistical methods

The analysis was based on the “intention-to-treat” principle. Baseline variables were calculated as means for continuous variables (age, level of disability, etc.) and as proportions for categorical (education level, diagnosis, etc.) and dichotomous variables (sex, presence or absence of alcohol or substance abuse, etc.). To test for differences between the intervention group and control group at baseline, the chi^2^ test was applied for categorical variables and the t-test for independent groups was applied for ordinal/continuous variables. All primary and secondary outcomes were calculated and presented as mean scores with standard errors (SE) at baseline and post intervention. Differences in means and proportions were presented with a 95% confidence interval and a *p*-value. The two-sided significance level for statistical tests was 5%. Differences between the intervention group and control group were analyzed using ANOVA to determine statistical significance. Multiple multivariate imputation was used to handle missing values. All covariates of supposed prognostic significance (variables theoretically associated with the outcome and variables predictive for missing data) were applied to impute a distribution of missing data [43]. The IBM SPSS Statistic version 10 for Windows was used for the statistical analysis.

Post hoc, we performed three additional analyses. First, we looked at within-group changes, comparing post-intervention assessments to baseline in the intervention group and the control group and using the paired t-test for statistical significance. Second, we examined differences in treatment effects when MA&R was delivered under “normal conditions” vs. during the COVID-19 pandemic. Participants were divided into two subsets: those who had completed post-intervention follow-up assessment before the COVID-19 lockdown, on the 12^th^ of March 2020, and those who had completed follow-up during the lockdown. Within these two subsets, differences between the intervention group and the control group were analyzed, using the t-test for independent samples. Third, we examined differences in treatment effects when MA&R was delivered in different sectors. Participants were divided into two subgroups: Those who were recruited in community mental health centers and those who were recruited in municipal mental health centers. Within these two subgroups, differences between the intervention group and the control group were analyzed, using the t-test for independent samples.

### Modifications due to the COVID-19 pandemic

The trial was partly conducted during the COVID-19 pandemic (a timeline is shown in the supplementary Fig. 1). The COVID-19 restrictions affected the interventional context, namely by restricting opportunities for engaging in activities. Thus, we performed a post hoc analysis, as described above.

### Ethics

The trial was conducted in accordance with the Helsinki Declaration and approved by the Ethics Committee of the Capital Region, Copenhagen, Denmark (H-18017307) and the Danish Data Protection Agency (VD-2018–299, I-suite nr: 6543). The study protocol is registered at http://www.clinicaltrials.gov/ (Protocol Record NCT 03,963,245). Participants’ data were collected, stored, and processed following General Data Protection Regulation (GPDR). All participants gave informed written consent for their data to be used in the study.

## Results

### Participant flow

Between September 2018 and August 2020, 139 participants were enrolled and randomly allocated to MA&R in addition to standard mental health care (*n* = 70) or standard mental health care alone (*n* = 69). The number of participants included in the study exceeded the 128 participants needed according to the sample size calculation, because a minimum of four participants was required to start an MA&R group. Thus, recruitment proceeded until enough participants were enrolled to start an MA&R group at each study site. Post intervention assessments were conducted between April 2019 and June 2021. All questionnaires were completed by the participants (*n* = 139) at baseline. At post-intervention follow-up, 113 participants completed questionnaires providing data on at least the primary outcome (Questionnaires completed by participants at follow up: POES-S: *n* = 113, QPR: *n* = 113, WHODAS.2.0 12: *n* = 108, MANSA: *n* = 103, EuroQOL: *n* = 111); 26 participants were lost to follow-up.

**Fig. 2:**
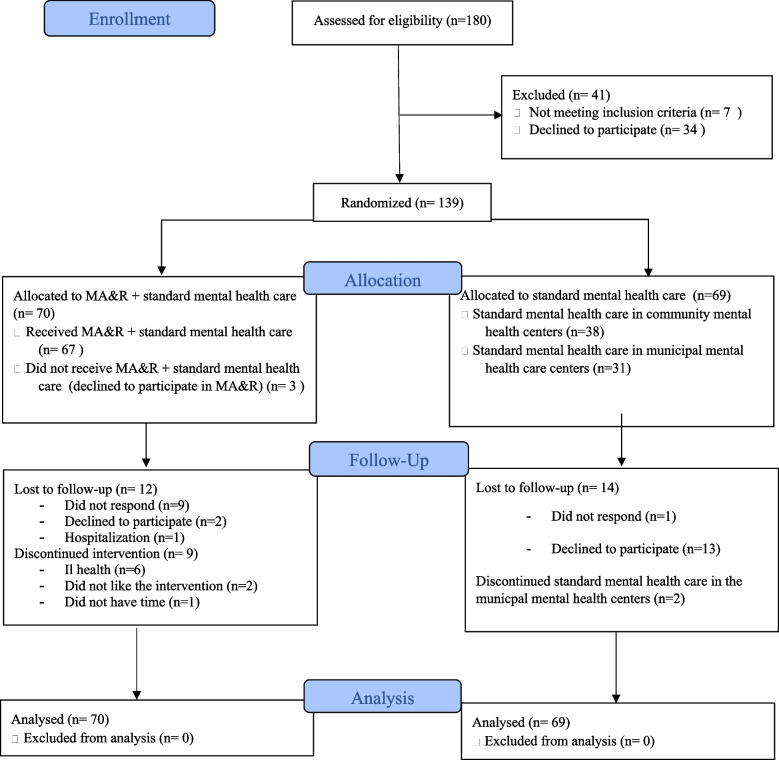
CONOSRT Flow diagram

No significant differences were found between responders and non-responders in age, sex, educational level, functioning measured using MINI ICF-APP or any of the baseline measures. A flow chart is depicted in Fig. 2.

### Baseline data

The trial included 71women and 68 men. The participants’ mean age was around 43,8 (SD 12,1). Most (*n* = 112, 80,6%) were single, did not have children (*n* = 97, 69,8%), and lived alone (*n* = 103, 74,1%). The mean MINI-ICF-APP Score was 19,3 (SD 5,2), which indicated marked disability. No statistical differences were found between groups with respect to demographic, clinical or baseline measures. A detailed description of characteristics of the study sample can be found in Table 2.

**Table 2:** Demographic characteristics

	MA&R + standard mental health care (*n* = 70)	Standard mental health care (*n* = 69)
Age, mean (SD)	42 (11,9)	44 (12,4)
**Gender, n (%)**		
Female	35 (50)	36 (52)
Male	35 (50)	33 (48)
**Marital status, n (%)**		
Single	56 (80)	56 (81)
Married or in a relationship	14 (20)	13 (19)
**Parental status, n (%)**		
Children	19	22
No children	51	46
**Employment status, n (%)**		
Employed	4 (6)	0 (0)
Studying	3 (4)	2 (3)
Unemployed	28 (40)	30 (43,5)
Receiving retirement or early retirement pension	27 (38,6)	31 (44)
Receiving sickness benefit	7 (10)	2 (2,9)
Unemployed and receiving no benefits	1 (1,4)	4 (5,8)
**Educational level, n (%)**		
Lower secondary education	19 (27,1)	14 (20,3)
Higher secondary education	19 (27,1)	19 (27,5)
Vocational training	10 (14,3)	13 (18,8)
University (bachelor’s degree)	10 (14,5)	16 (23,2)
University (master’s degree)	12 (17,1)	7 (10)
**Living status, n (%)**		
Living alone	49 (70)	54 (78,2)
Living with partner	13 (18,6)	8 (11,6)
Living alone with children under 18 years	3 (4,3)	3 (4,3)
Living with partner and children under 18 years	5 (7,1)	4 (5,8)
**Housing status, n (%)**		
Rented house/apartment	49 (70)	48 (69,6)
Owned house/apartment	15 (21,4)	11 (15,9)
Supported accommodation	5 (7,1)	10 (14,5)
**Diagnosis, n (%)**^a^		
Schizophrenia, schizotypal disorder, or psychosis	40 (57,1)	36 (52,2)
Depressive disorders	11 (15,7)	14 (20)
Bipolar disorders	11 (15,7)	10 (14,5)
Anxiety disorders	10 (14,3)	14 (20)
PTSD	5 (7,1)	7 (10)
Eating disorders	2 (2,9)	1 (1,4)
Personality disorders	7 (7)	9 (13)
Doesn`t know	6 (8,6)	4 (5,8)
**Alcohol or substance abuse, n (%)**		
Alcohol or substance abuse	5 (0,7)	3 (0,4)
No alcohol or substance abuse	65 (99,3)	66 (99,6)
**Functioning and disability, mean (SD)**		
Mini-ICF-APP Social functioning	19.81 (5,5)	18,91 (4,9)
Moderate disability^1^, n (%)	16 (22.9)	19 (27.5)
Marked disability^2^, n (%)	40 (57.1)	41 (59.4)
Severe disability^3^, n (%)	13 (18.6)	9 (13)
Extreme severe disability^4^, n (%)	1 (1.4)	0

*Abbreviations: SD* Standard deviations, *PTSD* Post-traumatic stress disorder, Footnote explanation: Distribution of disability severity, according to MINI ICF APP scores

^a^Total % > 100 because several participants reported more than one psychiatric diagnosis

^b^8-15 points

^c^16-24 points

^d^25-37 points

^e^38 to 52 points

Each site recruited between 5 and 19 participants and conducted one or two MA&R groups. More participants (about 60%) were recruited from the CMCHs than from the other sites. Between September 2018 and May 2021, a total of 10 MA&R groups were conducted and completed. As mentioned above, the MA&R groups conducted between March 2020 and May 2021 were affected by COVID-19 and related restrictions. Fidelity assessments were completed for all MA&R groups, showing good or optimal fidelity to MA&R. Fidelity scores for each recruitment site are presented in the supplementary table 1.

On average, participants in MA&R attended 15,4 sessions (SD: 5,7), corresponding to a mean of 7,0 (SD 3,2) group sessions and 8,3 (SD 2,9) one-on-one sessions; 14 (20%) participants attended 0–10 sessions, 33 (49%) attended 11–19 sessions, and 20 (29%) attended 20–22 sessions.

A total of 40 participants (59% of the participants starting MA&R) received individual support in addition to attending the planned sessions. Each of those participants had on average 2,8 encounters (SD 2,0) with either the occupational therapist or the peer worker.

As shown in Table 3, intention-to-treat analysis showed no significant difference at post-intervention follow-up between the intervention group and the control group on the primary outcome, activity engagement (1.1, 95% CI: -1,9, 3,3, *p* = 0.315). Nor did the groups differ with regard to functioning, personal recovery, or quality of life (Table 3). For safety measures, no between-group differences were found in psychiatric admissions, bed days, in somatic admissions or bed days, as shown in the supplementary table 2.

**Table 3:** Descriptive and inferential analysis of MA&R + standard mental healthcare and standard mental health care at baseline (T0) and at post intervention follow up (T1)

	MA&R + standard mental healthcare				Standard mental health care						
**Outcome measures** **↓/↑ indicates preferable outcome↑**	**T0**	**T1**	**T0-T1 Within group**	***p*-value**	**T0**	**T1**	**T0-T1 Within group**	**P**	**T1-T1** **Between groups**	***p*-value**	***d***
	**Mean (SD) (SEM)**	**Mean (SD) (SEM)**	**Est. (95% CI)**		**Mean (SD), (SEM)**	**Mean (SD) (SEM)**	**Est (95% CI)**		**Est (95%CI)**		
*Occupational engagement*											
Profiles of Occupational Engagement in People with Severe Mental Illness – Self report **↑**	21.1 (5) (0.6)	23.7 (5.9) (0.7)	2.5 (1.4, 3.7)	0.00	21.1 (5) (0.6)	22.5 (6.6) (0.8)	1.4 (0, 2.8)	0.04	1.1 (-1.0, 3.3)	0.3	0.19
*Functioning*											
WHODAS 2.0 12 item ↓	31.2 (6.7) (0.8)	29.4 (10.1) (1.2)	-1.7 (0.5, -4.1)	0.13	29.5 (6.6) (0.8)	27.5 (7.5) 0.9)	-2 (-0.3, -3.7)	0.01	1.9 (-1.1, 5.)	0.2	0.21
WHODAS 2.0 11 item (without work item) ↓	28.4 (6.7) (0.8)	26.9 (9.2) (1.1)	-1.5 (0.5, -3.6)	0.15	27.1 (6.6) (0.8)	25.0 (6.6) (0.8)	-2. (-0.4, -3.6)	0.01	1.84 (-0.9, 4.6)	0.2	0.24
WHODAS 2.0 36 item ↓	56.2 (17.6) (2.1)	50.2 (26) (3.1)	-6 (-0.3, -11.6)	0.03	53.7 (20) (2.4)	46.9 (23.2) (2.8)	-7.3 (-2, -12.6)	0.00	3.3 (-4.8, 11.5)	0.42	0.19
WHODAS 2.0 domain score *Cognition ↓*	14.8 (4.2) (0.5)	13.82 (5) (0.6)	-0.9 (0.2, -2.2)	0.12	14.3 (3.3) (0.4)	13.2 (4.2) (0.5)	-1.1 (0.1, -2.3)	0.07	0.6 (-1, 2.3)	0.44	0.04
WHODAS 2.0 domain score Mobility ↓	10.4 (3.4) (0.4)	9.9 (4.2) (0.5)	-0.5 (0.6, -1.7)	0.38	9.2 (3.3.) (0.4)	8.9 (3.3) (0.4)	-0.2 (05, -0,9)	0.54	1,0 (-0,4, 2,5)	0,.17	0.04
WHOADS 2.0 domain score Self-care ↓	7.8 (2.5) (0.3)	7.5 (3.4) (0.4)	-0.2 (0.5, -1.)	0.56	7.4 (2.5) (0.3)	7.2 (2.5) (0.3)	-0.2 (0.5, -1)	0.51	0.3 (-0.7, 1.4)	0.53	0.1
WHODAS 2.0 domain score Getting along* ↓*	14.4 (3.4) (0.4)	14.1 (5) (0.6)	-0.3 (0.6, -1.3)	0.53	14 (4.2) (0.5)	12.5 (5) (0.6)	-1.5 (-0.3, -2.7)	0.01	1.5 (0, 3.2)	0.06	0.3
WHODAS 2.0 domain score Life activities (Household)↓	12.2 (4.2) (0.5)	11.1 (5) (0.6)	-1 (0.1, -2.2)	0.07	12.1 (3.3) (0.4)	10.8 (4.2) (0.5)	-1.4 (-0.3, -2.4)	0.00	0.2 (-14, 1.9)	0.74	0.04
WHODAS 2.0 domain score Participation↓	24 (5) (0,6)	22 (6.7) (0.8)	-1.9 (-0,4, -3.3)	0.01	23.6 (5) (0.6)	21.1 (6.6) (0.8)	-2.5 (-1, -4,1)	0.00	0.9 (-1.2, 3.2)	0.4	0.13
*Personal recovery*											
Questionnaire about Recovery Processes 15 ↑	32.2 (10.9) (1.3)	36.6 (13.4) (1.6)	4.37 (1.9, 6.7)	0,00	32.3 (10) (1.2)	35 (10.8) (1.3)	2.7 (-0.4, 5.8)	0.08	1.56 (-2.5, 5.7)	0.46	0.13
*Quality of life*											
Manchester Short Assessment of Quality of Life ↑	47.7 (10,1) (1.2)	51.2 (12.6) (1.5)	3.2 (0.6, 5.8)	0.01	46. 3 (10.8) (1.3)	50.8 (12.5) (1.5)	4.3 (1.3, 7.2)	0.00	0.4 (-3.8, 4.7)	0.84	0.03
EQ-5D-5L: EuroQol Five Dimensions Questionnaire with Five Levels ↑	0.6 (0.17) (0.02)	0.6 (0.25) (0.03)	0.007 (-0.04, 0.06)	0.76	0.63 (0.17) (0.02)	0.64 (0.17) (0.02)	0.01 (-0.03, 0.06)	0.42	0.02 (-0.10. 0.49)	0.49	0.09
EQ-5D-5L Health index	53.6 (1.7) (0.2)	58 (25.2) (3)	4.4 (-1.4, 10.3)	0.13	55.5 (1.7) (0.2)	58.3 (23.2) (2.8)	2.7 (-3.6, 9.1)	0.4	0.2 (-8.4, 7.9)	0.95	0.01

*Abbreviations*: *SD* Standard deviations, *SEM* Standard error of the mean, *CI* Confidence Interval, *d* Cohen`s *d*

Post hoc analysis showed that both groups improved in activity engagement and quality of life. Both groups improved in functioning measured using the 36 items in WHODAS 2.0; when using the 12 items in WHODAS, only improvement in the control group was significant. The control group showed significant improvement in the *getting along* and *household* domains. Both the intervention group and the control group improved in the *participation* domain. Only the intervention group improved in personal recovery. No improvements were found in health-related quality of life in any of the groups (Table 3).

When comparing the subsets of participants completing post-intervention follow-up, before and after the COVID-19 lockdown, we found a between-group difference of 2.8-points favoring the intervention group (*p* = 0.064) before the COVID lockdown, and almost no difference during lockdown (Table 4). The lack of differences between groups during COVID 19 was ascribed to the finding that the control group performed better on the POES-S scale during lockdown.

**Table 4:** Mean scores on POES-S among subsets of participants completing post-intervention assessment before and during COVID 19 lockdown. Footnotes explanation

	MA&R + standard mental health care (SD)	Standard mental health care (SD)	Est (95% CI)	*p*-value
Before COVID 19 lockdown^a^	23.77 (6)	20.81 (5.3)	2.85 (-0.16. 5.88)	0.064
After COVID 19 lockdown^b^	23.94 (5.9)	23.68 (6)	0.1 (-2.98. 3.19)	0.94

^a^Comprises 41.7% of the sample

^b^Comprises 58.3% of the sample

## Discussion

To our knowledge, this is the first trial to evaluate the effectiveness of a co-led peer occupational therapy intervention combining group sessions with individual sessions and the option of individual support. Findings from the trial did not confirm our study hypothesis, as MA&R was not superior to standard mental health care in improving activity engagement, personal recovery, functioning or quality of life. We still consider it important to study activity engagement, since occupational therapy services are delivered in institutional facilities but target services users` engagement in everyday life outside the institution. It is therefore critical to develop interventions that address the challenges of transferring gains from interventional settings to meaningful everyday life activities in the community [25, 44, 45]. Moreover, potential explanations for the null findings should be considered.

First, the differences between the RCT arms may have been smaller than anticipated. The control condition in this study, care as usual, may explain why MA&R was not superior, since some of the core components in MA&R were also available in standard mental health care. For instance, peer support and occupational therapy were part of standard mental health care in several sites and therefore available to the control group, too.

Another potential explanation concerns the strategy to support transfer of gains (skills, competences) acquired during sessions to activity engagement in the community. Contrary to the BEL intervention, MA&R did not incorporate home assignments to be practiced in real life conditions. Instead, MA&R offered individual support, but this component of the intervention was optional. Thus, the utilization of individual support was much lower than we anticipated when designing MA&R. While COVID-19 may partly explain the low uptake of individualized support and transition of activities in the community, the low use of support may also be a result of implementation failure. Perhaps the individual support component was too vaguely described in the MA&R manual. The manual emphasized that support should be flexible and tailored to the participants’ preferences. Within occupational therapy literature, conceptual practice models exist [46–48] that could have guided the MA&R providers in providing support to enable activity engagement. Offering more detailed descriptions of how to provide such tailored support may potentially have enhanced delivery and, consequently, uptake of support. Identifying individual support as a facilitator for engaging in activities in the community, the study informing the development of MA&R was based on participants’ experiences from Individual Placement and Support, a rehabilitation intervention designed to help people with mental illness pursue and obtain vocational goals [25]. Although the IPS intervention is useful for enabling participation in work and education, it may not be applicable in other areas of everyday life. However, little is known about how people with psychiatric disabilities experience support to enable participation in a broader spectrum of daily activities relating to the household, social life, hobbies, and interests, etc. We therefore suggest that individual support, as a component of MA&R, should be further developed within a theoretical framework and in collaboration with people with lived experiences of receiving such services as part of their recovery process. COVID-19 may also have affected overall intervention delivery and the intended impact of the intervention, as the restrictions impaired some of our key change mechanisms, for instance limiting the range of available meaningful activities or reducing access to peer support because of social distancing. Moreover, due to COVID-19 regulations, the MA&R groups starting during COVID consisted of 4 to 5 participants, whereas MA&R groups typically included 5 to 9 participants before the lockdown. The small group size became problematic if only a few participants were absent or decided to discontinue the intervention. Post hoc analysis suggested that MA&R may have been better than standard mental health services before, but not during, the lockdown, but it is important to keep in mind that these subgroup analyses lack statistical power and are not based on enough participants. However, the POES-S scores in the control group before vs. during the COVID-19 do not clearly support the hypothesis of restrictions impeding activity engagement. Thus, the change mechanisms relating to meaningful activity engagement should be investigated in future studies and inform intervention refinements. Future research should examine the effects of activity engagement interventions over a longer period of time, for instance by extending the primary outcomes to 3 or 6 months after the intervention has ended.

As this RCT was a multicenter trial, between-group comparisons may have been blurred by differences in standard mental health care across sites. To explore this further, we conducted a between-group comparison of activity engagement, personal recovery, functioning, and quality of life, stratified by type of services (CMCHs vs. municipal mental health services). However, the post hoc analysis did not show substantial differences in treatment effects between the CMCHs and the municipal health services. Results from the analysis are presented in the supplementary table 3 and 4.

### Methodological considerations

This study has several strengths: a sample size calculation was performed prior to recruitment; the trial reached the intended number of participants; randomization was conducted with adequate allocation concealment; blinding of researchers was obtained during analysis and while writing the conclusion; all outcomes are reported; analysis was based on intention-to-treat analysis; and MA&R was delivered with good and optimal fidelity and with relatively low attrition rates. The study also has limitations: it was not possible to blind participants and staff to intervention allocation, and this increased the risk of expectation and collateral intervention bias [49]; the outcome measures consisted of self-report instruments, which are more prone to bias than assessor-rated or objective measures; and outcomes were measured only twice, at baseline and post-intervention follow-up. MA&R is a complex intervention with multiple interacting components, and two measurement time points were not sufficient to show whether some modes or components, such as the high intensity mode in MA&R I vs. the low intensity mode in MA&R II, were more effective than others. The trial was partly conducted during the COVID-19 pandemic, and the results may be unique to this context, thus limiting the study`s external validity when generalizing the findings to “normal conditions”. We did not obtain specific data on participants’ service use with respect to standard mental care, other than the use of psychiatric and somatic inpatient services, like admissions. In this study, standard mental health was broadly defined, and we do not have information about what type of care participants received. This lack of information leads to a lack of clarity, for example concerning the comparator and the treatment–control contrast [50, 51].

## Conclusion

The trial did not provide evidence that MA&R, a co-led peer occupational therapy intervention, is superior to standard mental health care delivered in the CMCHs, ASSCs or rehabilitation teams in terms of promoting activity engagement, personal recovery, functioning or quality of life.

For now, we cannot recommend that MA&R is implemented in mental health practice. The lockdown might have affected intervention delivery, and impeded its potential impacts, therefore, we recommend that MA&R is re-evaluated under “normal conditions”. Before a new evaluation, we furthermore recommend an update of the intervention based on the gathered knowledge.

## Supplementary Information

**Additional file 1:** **Supplementary figure 1.** Timeline. Study periodbefore and during the COVID 19 lockdown.

**Additional file 2:** **Supplementary table 1.** A overview over fidelityscores and included participants, at the recruitment sites: Community mentalhealth center = CMCH, Municipalities mental health services = MMHS. Explanationto fidelity score: Below 25 points means no fidelity to MA&R, between 26and 32 points reflects OK fidelity to MA&R, between 33 and 38 indicatesgood fidelity to MA&R and between 39 and 42 is optimal fidelity toMA&R.

**Additional file 3: Supplementary table 2.** Harms.

**Additional file 4:** **Supplementary table 3.** Between-group differencesin mean scores of POES-S, WHODAS 2.0 11 item (without work item), QPR and MANSAat post intervention follow up, in the municipal mental health services group.** Supplementary table 4.** Between-groupdifferences in mean scores of POES-S, WHODAS 2.0 11 item (without work item),QPR and MANSA at post intervention follow up, in the community mental healthcenter group.

## Data Availability

Danish legislation prevents us from publicizing the datasets generated and/or analyzed during the current study, but they can be provided by the corresponding author on reasonable request.
