# Supplementary material for: Meaningful Activities and Recovery (MA&R): a co-led peer occupational therapy intervention for people with psychiatric disabilities. Results from a randomized controlled trial
Source: BMC Psychiatry. 2023 Jun 6;23:406. doi: 10.1186/s12888-023-04875-w (PMC10243265; doi:10.1186/s12888-023-04875-w)
Supplement: Supplementary file 1 — Additional file 1: Supplementary figure 1. Timeline. Study periodbefore and during the COVID 19 lockdown. [file 12888_2023_4875_MOESM1_ESM.docx]

Supplementary figure 1: Timeline. Study period before and during the COVID 19 lockdown

2021

2020

Post interventions follow up assessments

2019

2018

Covid 19 lockdown

Recruitment and baseline assessments
