# Supplementary material for: Meaningful Activities and Recovery (MA&R): a co-led peer occupational therapy intervention for people with psychiatric disabilities. Results from a randomized controlled trial
Source: BMC Psychiatry. 2023 Jun 6;23:406. doi: 10.1186/s12888-023-04875-w (PMC10243265; doi:10.1186/s12888-023-04875-w)
Supplement: Supplementary file 2 — Additional file 2: Supplementary table 1. A overview over fidelityscores and included participants, at the recruitment sites: Community mentalhealth center = CMCH, Municipalities mental health services = MMHS. Explanationto fidelity score: Below 25 points means no fidelity to MA&R, between 26and 32 points reflects OK fidelity to MA&R, between 33 and 38 indicatesgood fidelity to MA&R and between 39 and 42 is optimal fidelity toMA&R. [file 12888_2023_4875_MOESM2_ESM.docx]

| Recruitment site | Number of participants in intervention group | Number of MA&R groups | Fidelity score for group 1 | Fidelity score for group 2 |
| --- | --- | --- | --- | --- |
| CMHC 1 | 19 | 2 | 39 | 42 |
| CMHC 2 | 17 | 2 | 36 | 42 |
| CMHC 3 | 5 | 1 | 42 | N/A |
| MMHS 1 | 8 | 1 | 37 | N/A |
| MMHS 2 | 9 | 2 | 39 | 42 |
| MMHS 3 | 10 | 2 | 37 | 41 |

Supplementary table 1: A overview over fidelity scores and included participants, at the recruitment sites: Community mental health center = CMCH, Municipalities mental health services = MMHS*. Explanation to fidelity score*: Below 25 points means no fidelity to MA&R, between 26 and 32 points reflects OK fidelity to MA&R, between 33 and 38 indicates good fidelity to MA&R and between 39 and 42 is optimal fidelity to MA&R.
