# Supplementary material for: Meaningful Activities and Recovery (MA&R): a co-led peer occupational therapy intervention for people with psychiatric disabilities. Results from a randomized controlled trial
Source: BMC Psychiatry. 2023 Jun 6;23:406. doi: 10.1186/s12888-023-04875-w (PMC10243265; doi:10.1186/s12888-023-04875-w)
Supplement: Supplementary file 3 — Additional file 3: Supplementary table 2. Harms. [file 12888_2023_4875_MOESM3_ESM.docx]

| Variable | Incident Rate Ratio (SE) | P-value | 95 %CI |
| --- | --- | --- | --- |
| ***Psychiatric bed days*** |  |  |  |
| Standard mental health care (ref) | 1 |  |  |
| MA&R + Standard mental health care | 1,2 (1) | 0,82 | 0,24-6,19 |
| ***Psychiatric Admissions*** |  |  |  |
| Standard mental health care (ref) | 1 |  |  |
| MA&R + Standard mental health care | 0,84 (0,36) | 0,69 | 0,36-1,96 |
| ***Somatic bed days*** |  |  |  |
| Standard mental health care (ref) | 1 |  |  |
| MA&R + Standard mental health care | 5,77 (6,89) | 0,14 | 0,55-60 |
| ***Somatic admissions*** |  |  |  |
| Standard mental health care | 1 |  |  |
| MA&R +standard mental health care | 0,69 (0,42) | 0,55 | 0,21 – 2,27 |

Supplementary table 2: Harms
