# Supplementary material for: Meaningful Activities and Recovery (MA&R): a co-led peer occupational therapy intervention for people with psychiatric disabilities. Results from a randomized controlled trial
Source: BMC Psychiatry. 2023 Jun 6;23:406. doi: 10.1186/s12888-023-04875-w (PMC10243265; doi:10.1186/s12888-023-04875-w)
Supplement: Supplementary file 4 — Additional file 4: Supplementary table 3. Between-group differencesin mean scores of POES-S, WHODAS 2.0 11 item (without work item), QPR and MANSAat post intervention follow up, in the municipal mental health services group. Supplementary table 4. Between-groupdifferences in mean scores of POES-S, WHODAS 2.0 11 item (without work item),QPR and MANSA at post intervention follow up, in the community mental healthcenter group. [file 12888_2023_4875_MOESM4_ESM.docx]

| Outcome measure | Est (95 % CI) | P-value |
| --- | --- | --- |
| Profiles of occupational engagement in People with Severe Mental Illness – Self report (POES-S) | 1.2 (-2, 4.4) | 0.5 |
| WHODAS 2.0 (without work item) | -1.5 (-5.7, 2.5) | 0.5 |
| Questionnaire about Recovery Processes (QPR) | 1.8 (-4.9, 7) | 0.7 |
| Manchester Short Assessment of Quality of Life (MANSA) | 1 (-4.8, 6.9) | 0.8 |

Supplementary table 3: Between-group differences in mean scores of POES-S, WHODAS 2.0 11 item (without work item), QPR and MANSA at post intervention follow up, in the municipal mental health services group.

| Outcome measure | Est (95 % CI) | P-value |
| --- | --- | --- |
| Profiles of occupational engagement in People with Severe Mental Illness – Self report (POES-S) | 1.8 (-0.75, 4.5) | 0.2 |
| WHODAS 2.0 (without work item) | -1.6 (-5.3, 2.) | 0.4 |
| Questionnaire about Recovery Processes (QPR) | 2.7 (-2.7, 8.1) | 0.3 |
| Manchester Short Assessment of Quality of Life (MANSA) | 0.6 (-5.3, 6.4) | 0.9 |

Supplementary table 4: Between-group differences in mean scores of POES-S, WHODAS 2.0 11 item (without work item), QPR and MANSA at post intervention follow up, in the community mental health center group.
